# The Roles of Sirtuin Family Proteins in Cancer Progression

**DOI:** 10.3390/cancers11121949

**Published:** 2019-12-05

**Authors:** Erhu Zhao, Jianbing Hou, Xiaoxue Ke, Muhammad Nadeem Abbas, Saima Kausar, Lei Zhang, Hongjuan Cui

**Affiliations:** 1 State Key Laboratory of Silkworm Genome Biology, College of Biotechnology, Southwest University, Chongqing 400716, China; erhuzhao@126.com (E.Z.); houjb7905@163.com (J.H.);; 2 Medical Research Institute, Southwest University, Chongqing 400716, China; 3 Engineering Research Center for Cancer Biomedical and Translational Medicine, Southwest University, Chongqing 400716, China

**Keywords:** Sirtuins, cell viability, apoptosis, metastasis, tumorigenesis

## Abstract

Sirtuin family members are characterized by either mono-ADP-ribosyltransferase or deacylase activity and are linked to various cancer-related biological pathways as regulators of transcriptional progression. Sirtuins play fundamental roles in carcinogenesis and maintenance of the malignant phenotype, mainly participating in cancer cell viability, apoptosis, metastasis, and tumorigenesis. Although sirtuin family members have a high degree of homology, they may play different roles in various kinds of cancer. This review highlights their fundamental roles in tumorigenesis and cancer development and provides a critical discussion of their dual roles in cancer, namely, as tumor promoters or tumor suppressors.

## 1. Overview of the Sirtuin Family

**Figure 1:**
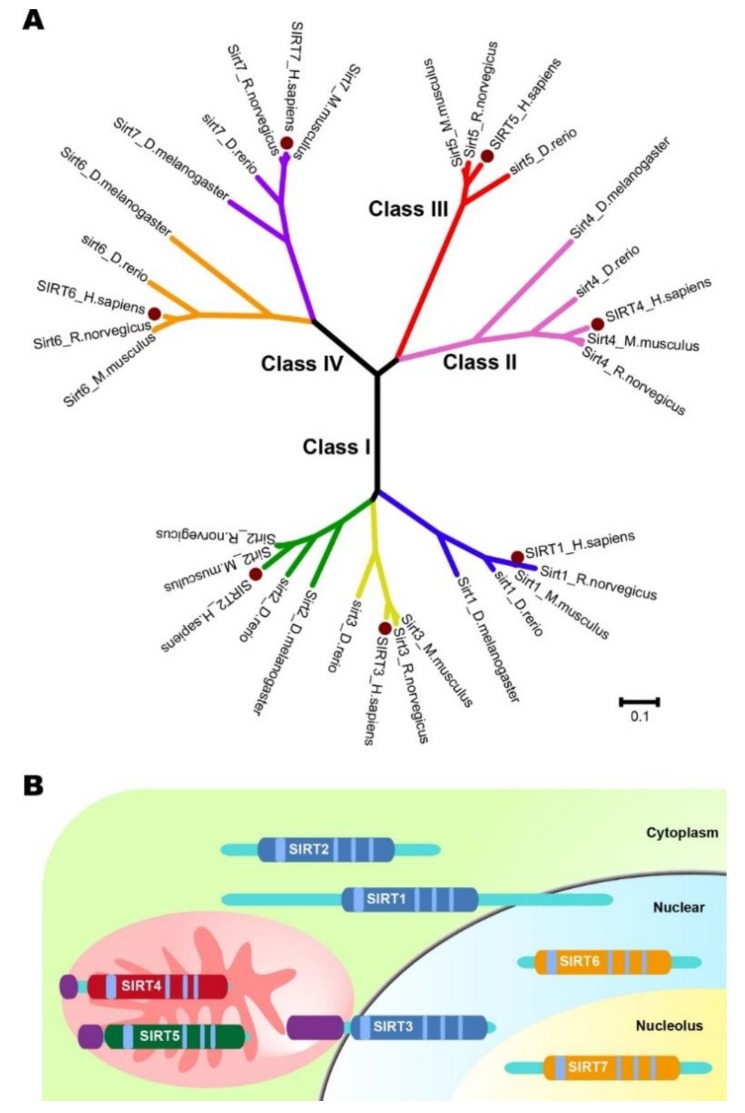
Human sirtuin protein family. (**A**) Phylogenetic tree of sirtuins family. The clustering analysis was constructed based on the full-length amino acid sequences of sirtuins in different species, including *Drosophila melanogaster*, *Danio rerio*, *Mus musculus*, *Rattus norvegicus*, *and Homo sapiens*. Sirtuin protein family are shown in blue (sirtuin 1), green (sirtuin 2), yellow (sirtuin 3), pink (sirtuin 4), red (sirtuin 5), orange (sirtuin 6), and purple (sirtuin 7). They are divided into four groups: Class I (sirtuin 1, sirtuin 2, and sirtuin 3), Class II (sirtuin 4), Class III (sirtuin 5), and Class IV (sirtuin 6 and sirtuin 7). The human sirtuins are labeled with brown dots. (**B**) Schematic structure of the human sirtuins. Catalytic domains reflecting classes of sirtuins are shown in Blue (class I), Red (class II), Green (class III), and Orange (class IV); mitochondrial targeting sequences are shown in Purple; NAD+ binding regions are shown in a sky blue shade; The peptide chains are shown as light blue lines.

Sirtuins refer to a protein family that is highly conserved. Sirtuins exert mono-ADP-ribosyltransferase and deacylase activity. These proteins, due to the feature of a sequence in the ancestral yeast gene *silent information regulation 2* (*Sir2*) [1,2,3], were initially identified as silent mating factors. Mammalian sirtuins are homologues of yeast Sir2 proteins. Seven sirtuins (SIRT1–7) have been defined in human cells thus far. They collectively constitute the protein deacetylases in class III [4]. Functioning as NAD^+^-dependent protein deacetylases and/or ADP-ribosyltransferases, they are located in different subcellular compartments. Sirtuins may thus be distinguishable by their diverse subcellular localizations. SIRT1, SIRT6, and SIRT7 are mainly located in the nucleus [5,6], while SIRT3, SIRT4, and SIRT5 are mainly located in the mitochondria. SIRT1 and SIRT5 have been found in the cytoplasm [3,7]. SIRT2 is largely cytosolic but is able to translocate to the nucleus during mitosis [3,8]. Moreover, proteins in the SIRT family possess a conserved domain for core catalysis. Based on this feature, sirtuin proteins are divided into four classes phylogenetically, where SIRT1, SIRT2, and SIRT3 are grouped into class I, SIRT4 is grouped into class II, SIRT5 is grouped into class III, and SIRT6 and SIRT7 are grouped into class IV (Figure 1) [9].

Despite the SIRT family proteins initially being described as mono-ADP-ribosyltransferases, the SIRT family was later found to be able to deacetylate histone proteins in the presence of nicotinamide adenine dinucleotide^+^ (NAD^+^) [10]. By transferring the acetyl group to the adenosine diphosphate (ADP)-ribose moiety, they can combine lysine deacetylation with NAD+ hydrolysis to form 2′-O-acetyl-ADP-ribose and then release free nicotinamide (NAM), a feedback inhibitor of sirtuins [11,12,13]. The seven human sirtuins share an ~275 aa catalytic core that contains two domains: A smaller zinc-binding domain and a larger Rossmann fold domain. Together, the two domains form a specific structure to unite NAD^+^ as a cofactor [14].

Sirtuins, in virtue of their catalytic activity, are involved in various important biological processes, such as aging, the stress response, viability, differentiation, metabolism, apoptosis, and cell survival [3,11,14]. For years, the sirtuins have been under investigation in the scientific community, and some progress has been made. Despite these efforts, determining their complex roles in cancers remains a highly difficult challenge due to their dual characteristics as both tumor promoters and tumor suppressors revealed in different cancers. As a result of this characteristic, sirtuins have become increasingly important and attractive to researchers. In this paper, we focus on their seemingly dichotomous roles in cell viability, apoptosis, metastasis and tumorigenesis.

## 2. Sirtuins and Viability in Cancers

**Figure 2:**
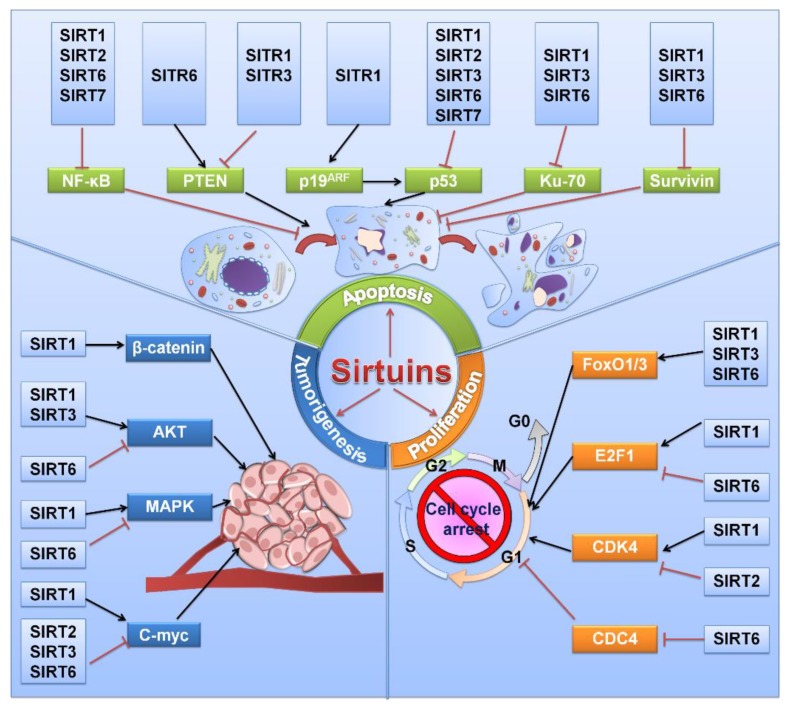
Roles of sirtuins in cell proliferation, apoptosis, and tumorigenesis.

Previous evidence has suggested the inhibitory effect of sirtuins on the viability of different tumor malignancies [15,16,17]. Sirtuins play complex and important roles regulating cancer cell growth and proliferation (Figure 2 and Table 1). The role of SIRT1 in cell viability is contradictory and complicated [18]. On the one hand, it maintains genetic stability in normal cells and decelerates cell growth and proliferation in some mouse models [19,20]. A decrease in SIRT1 in breast cancer is correlated with BRCA1 mutations, which indicates the role of SIRT1 as a tumor suppressor [21]. On the other hand, SIRT1 promotes cell growth and proliferation in most cancers, such as leukemia and thyroid and colorectal cancers [22,23,24]. As reported by Sasca et al., pharmacologic- or RNA interference (RNAi)-mediated SIRT1 inhibition reduced cell growth by restoring P53 activity [25]. In addition, SIRT1 also possesses the ability to deacetylate other proteins, such as FOXO3a, RB1, KU70 and E2F1, to facilitate cell growth [26,27,28,29]. Multiple studies have shown the process of SIRT1 being targeted by miR-22, miR-34a, miR-200a, miR-138, miR-30e-5P, miR-204, miR-212, and miR-449a to suppress cell proliferation in tumor progression [30].

Similar to SIRT1, the impact of SIRT2 on cell viability appears to depend on the cellular context or the kind of tumor. A number of studies have suggested the role of SIRT2 as a tumor suppressor that deacetylates a variety of substrates, including histone H4K16, P53, P65, FOXO1, FOXO3, and CDK4 [37,39,40,86,87,88]. Moreover, SIRT2 can interact with β-catenin and KDM4A to inhibit cell growth [89,90]. However, SIRT2 is also defined as an oncogenic factor associated with cell proliferation and shortened overall survival in pancreatic cancer, hepatocellular carcinoma (HCC), and neuroblastoma [36,38,42].

A major deacetylase in mitochondria, SIRT3, plays a crucial role in the regulation of cancer cell growth. Based on the kind of cancer and probably the statuses of the intracellular signaling pathways, it may function as a tumor promotor or a tumor suppressor. Recent studies have noted the regulation of SIRT3-mediated deacetylation on a variety of substrates, such as P53, GSK-3β, PDHA1, IDH2, and NMNAT2 [46,91]. It has been reported that SIRT3 can increase the ubiquitination and degradation of the oncoprotein MYC and inhibit prostate cancer progression both in vitro and in vivo [47].

There are reports that SIRT4 functions as a tumor suppressor, restraining the growth and proliferation of cells [56,92,93,94]. SIRT4 can induce G1 cell cycle arrest by inhibiting phosphorylated extracellular signal-regulated kinase, cyclin D and cyclin E in gastric cancer [92]. Moreover, SIRT4 suppresses the malignant progression of non-small cell lung cancer (NSCLC) via ERK-Drp1 pathway-mediated mitochondrial dynamics [57].

As a potential oncogene, SIRT5 mediates lysine deglutarylation, desuccinylation, and demalonylation. Wang et al. reported that SIRT5 impeded cell growth and functionally activated glutamate dehydrogenase 1 (GLUD1), a key regulator of cellular glutaminolysis, by both directly interacting with GLUD1 and leaving it deglutarylated [62]. Studies have demonstrated that SIRT5 can desuccinylate SHMT2, PKM2 and SOD1 and regulate their activities to stimulate cell proliferation [63,64,95]. However, few studies have discussed the mechanism of SIRT5-catalysed demalonylation in the progression of cancer cells.

Similar to other sirtuins, SIRT6 also plays a role in tumor suppression or progression. Various studies have demonstrated that SIRT6 can deacetylate several cancer-related genes, such as PKM2, NF-κB, HIF1α, CtBP, and JUN, which results in a reduction in cell proliferation [71,74,76,96,97]. Low SIRT6 expression has been reported in pancreatic cancer, colorectal cancer, and HCC [77,79]. However, several recent studies have reported that SIRT6 functions as a tumor promoter in other human cancers, such as skin squamous cell carcinoma, papillary thyroid cancer and acute myeloid leukemia (AML) [72,98,99]. SIRT6 promotes COX-2 expression by inhibiting AMPK signaling, thereby increasing cell proliferation and survival in the skin epidermis. In papillary thyroid cancer, SIRT6 promotes tumorigenesis by enhancing HIF-1α stability and prolonging its protein half-life [98]. Moreover, SIRT6 upregulation rescues the suppressive effect of LINC00319 (a long noncoding RNA, lncRNA) on AML cell growth [99].

Previous studies have also indicated that the deacetylation substrates of SIRT7, such as P53, H3K18, PAF53, NPM1, and GABP-β1, are critical mediators in multiple cellular activities [100]. SIRT7 can decrease the level of H3K18ac at the CDC4 promoter region to downregulate CDC4 expression and promote osteosarcoma cell proliferation [80]. Similar to other sirtuins, SIRT7 also functions as a tumor suppressor for cell growth. In oral squamous cell carcinoma (OSCC), SIRT7 can inhibit cell growth by promoting SMAD4 deacetylation [81].

## 3. Sirtuins and Apoptosis in Cancers

Apoptosis is triggered and controlled by counterbalancing pro- and antiapoptotic-associated genes such as BAX, BAK1, and BCL2 in response to various physiologic stresses [101,102]. Growing evidence shows that sirtuins are generally involved in apoptosis by regulating the expression of various components (Figure 2).

SIRT1 is predominantly located in the nucleus and acts as a regulator of apoptosis. In response to DNA damage and oxidative stress, SIRT1 exerts its antiapoptotic activity, deacetylating key apoptosis-related proteins and cell signaling molecules, such as P53, NF-κβ, FOXO3, KU70, AKT, MAPK, and NRF2 [103,104]. The upregulation of SIRT1 expression promotes apoptosis in K-Ras-driven lung adenocarcinoma [31]. SIRT1 negatively regulates the antiapoptotic gene survivin to trigger apoptosis through histone deacetylation at its promoter, which epigenetically silences survivin expression [21].

SIRT2 can antagonize P53-dependent transcriptional activation and induce apoptosis in response to DNA damage by catalyzing P53 deacetylation [105]. SIRT2 can promote the nuclear translocation of FOXO3a by binding to and deacetylating FOXO3a, which activates CASP8 and CASP3 and triggers cell apoptosis [106]. In cholangiocarcinoma, SIRT2 overexpression can inversely inhibit peroxidation-related apoptosis by activating MYC and increasing the production of antioxidants [41].

SIRT3 is primarily located in the mitochondrial matrix and possesses distinct biochemical activities and substrate specificities. The abnormal expression of SIRT3 induces apoptosis by affecting BAX, BCL2, and P53 in leukemia and lung cancer cells [49,53]. Conversely, SIRT3 also blocks apoptosis by deacetylating and negatively regulating AGFG1 downstream signaling in response to chemotherapeutic agents [52,107]. However, whether SIRT3 functions as a tumor promoter or suppressor in apoptosis remains controversial, and further studies will be needed to confirm this hypothesis.

There are few reports about the effects of SIRT4 and SIRT5 on cell apoptosis. SIRT4 plays a protective role in hypoxia-induced apoptosis by affecting Bax translocation [108]. However, SIRT4 silencing prevents the apoptosis of human colorectal cancer cells in response to 5-FU [109]. In addition, SIRT5 plays both antiapoptotic and antioxidative roles in neuroblastoma, and the overexpression of SIRT5 significantly protects neuroblastoma cells from staurosporine-induced apoptosis [68]. In hepatocellular carcinoma, SIRT5 can inhibit cell apoptosis by deacetylating cytochrome c [110].

SIRT6 overexpression induces massive apoptosis in cancer cells but not in normal cells. SIRT6 can drive apoptosis by deacetylating key cell signaling molecules, such as KU70, Bax, and P53, in response to DNA damage and oxidative stress [70,111,112].

In glioblastoma multiforme, SIRT6 induces cell apoptosis by inhibiting the JAK2/STAT3 signaling pathway [75]. SIRT6 promotes cell apoptosis by modulating the PTEN/AKT and ERK1/2 signaling pathways in colorectal and hepatocellular cancers [73,78].

As a major deacetylase, SIRT7 is able to deacetylate key cell signaling molecules, such as FOXO3 and DDB1, and thus regulates apoptosis progression in response to DNA damage and oxidative stress [113,114]. SIRT7 depletion also induces apoptosis by regulating the activity of MYC and subunits of the NF-κB family or through the mTOR/IGF2 and p38MAPK pathways to address various stimuli [82,83,115,116]. In addition, SIRT7 can regulate the expression of pro- and antiapoptotic genes by repressing miR-34a activity [84].

## 4. Sirtuins and Tumor Metastasis

**Figure 3:**
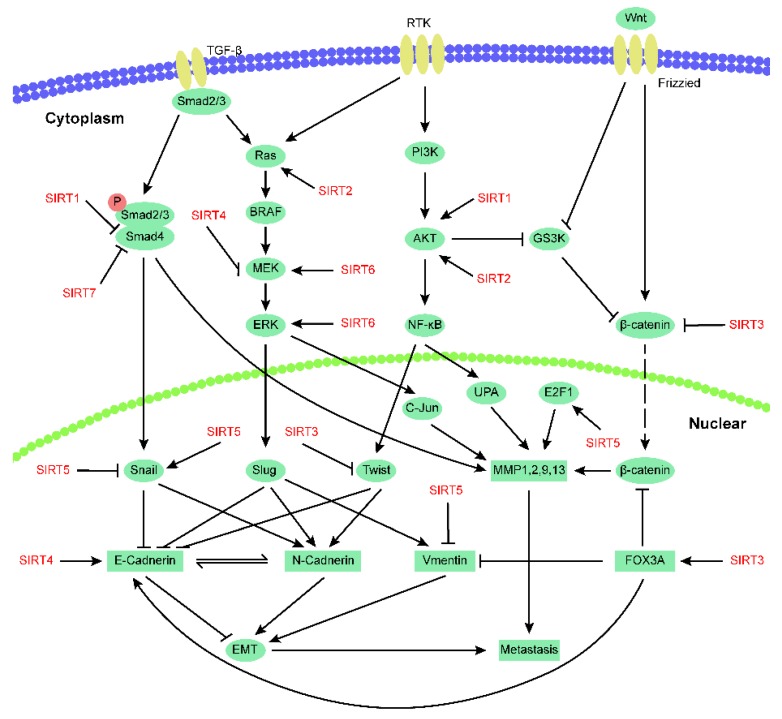
Contribution of sirtuins to cancer metastasis.

Tumor metastasis accounts for most cancer-related deaths worldwide and is a difficult challenge in cancer treatment. Researchers worldwide strive to understand the mechanisms involved in the migration and invasion of cancer. The functions of various sirtuins have been confirmed in tumor metastasis (Figure 3), which also play complex and important roles regulating cancer cell migration and invasion in different kinds of cancer (Table 1). Next, we will provide a basic overview of sirtuins and tumor metastasis.

The expression level of SIRT1 is related to tumor stage, tumor invasion, lymph node metastasis, and shortened overall survival in patients with gastric carcinoma [34]. Similarly, in breast cancer tissues and cells, SIRT1 is correlated with histological grade, tumor size, and lymph node metastasis. SIRT1 also enhances the activity of PI3K/AKT due to their direct interaction. Regarding AKT depletion, however, the SIRT1-mediated proliferative effect is only partially decreased in breast cancer [35]. In addition, the transcriptional level of SIRT1 is interrelated with lymph node-positive metastatic breast cancer [117]. Because of its relevance to lymph node status, stage, distant metastatic relapse, and P53 status in patients with triple-negative breast cancer (TNBC), SIRT1 expression is tightly correlated with a poor prognosis in TNBC and non-TNBC patients [118]. Moreover, the downregulation of SIRT1 attenuates the migration and invasion of prostate cancer cells. Such a relation highlights the possibility of SIRT1 as a promising target to preclude prostate cancer metastasis [119].

Experiments on nude mice have shown that SIRT1 promotes invasion and metastasis in breast cancer when it is weakly expressed, and the loss of SIRT1 in renal tubular epithelial cells exacerbates injury-induced kidney fibrosis [20]. By deacetylating Smad4 and lessening the impact of TGF-β signaling on MMP7, SIRT1 decreases epithelial to mesenchymal transition (EMT) in cancer and fibrosis [20]. Likewise, SIRT1 promotes EMT in prostate cancer cells by cooperating with the EMT-inducible transcription factor ZEB1. SIRT1 silencing restricts the expression of ZEB1. SIRT1 is recruited via ZEB1 to the E-cadherin proximal promoter, thereby deacetylating histone H3 and inhibiting the binding of RNA polymerase II and ultimately blocking the transcription of E-cadherin. Thus, SIRT1 acts as a positive regulator of EMT to influence the metastatic growth of prostate cancer cells, while SIRT1 overexpression serves as a potential therapeutic target to reverse EMT and defend against prostate cancer progression [17].

SIRT2 has been reported to reduce E-cadherin expression in mouse embryonic fibroblasts (MEFs) and was recently shown to positively regulate migration and invasion in the context of cancer [89]. SIRT2 is upregulated in cancer tissues relative to adjacent normal tissues in several kinds of cancer [43,44,120]. By deacetylating and activating protein kinase B (AKT), it can enhance EMT to target the AKT/GSK3β/β-catenin signaling pathway in hepatocellular carcinoma [43] and promote the migration and invasion of gastric cancer through the RAS/ERK/JNK/MMP-9 pathway [44].

Previous studies have shown the ability of SIRT3 to activate FOXO3A and then to suppress EMT and the migration and invasion of prostate cancer cells. SIRT3 can simultaneously suppress the Wnt/β-catenin pathway, thereby triggering EMT-associated processes [54]. As reported previously, SIRT3 controls the EMT process and metastatic motility of cancer cells through Twist in ovarian carcinoma [55]. In addition, SIRT3 has the capability to suppress cell migration and invasion in HCC and pancreatic ductal adenocarcinoma [50,51]. However, some conflicting reports have shown that the high expression of SIRT3 is interrelated with positive lymph node metastasis in breast cancer and disclosed an association between the levels of SIRT3 and lymph node metastasis [117]. Furthermore, SIRT3 deletion significantly represses the migration of colorectal cancer cells by reducing the transcription of metastatic-related genes, such as EGFR and BRAF [48].

SIRT4 inhibits NSCLC cell invasion and migration, perhaps affecting the invasive capability of cancer by hampering MEK/ERK activity [57]. A deficiency in SIRT4 facilitates liver tumor development and lung metastasis in mice with xenografts and Sirt4 knockout (Sirt4^-/-^) by promoting colony formation and migration and enhancing the sphere formation of hepatocellular cancer cells [61]. In colorectal cancer cells, SIRT4 suppresses migration and invasion while upregulating E-cadherin expression. Its expression weakens with the progression of invasion and metastasis, and a low expression level is correlated with a poor prognosis [59]. A seemingly important part of gastric cancer (GC), SIRT4, is involved in regulating EMT. A low expression level of SIRT4 is negatively correlated with tumor size, pathological grade, and lymph node metastasis and predicts a poor prognosis. SIRT4 also suppresses cell proliferation. It is responsible for the regulation of EMT, thereby regulating cell migration and invasion in GC [60].

SIRT5 contributes to cell invasion in HCC by targeting E2F1 [65]. Additionally, targeting SIRT5 enables miR-299-3p to inhibit the migration and invasion of HCC cells [69]. Some reports have noted the promoting effect of SIRT5 on cell migration by inducing Vimentin acetylation and enhancing EMT by upregulating Snail and downregulating E-cadherin in HCC [66].

SIRT6, another member of the sirtuin protein family, functions in multiple complex ways in cancer. SIRT6 expression enhancement is related to clinical and pathological parameters such as T and N classification in the tumors of NSCLC patients. SIRT6 overexpression not only strengthens the phosphorylation of extracellular signal-regulated kinase 1/2 (p-ERK1/2) but also activates MMP9 and promotes the migration and invasion of tumor cells. In contrast, the deletion of SIRT6 contributes to the metastasis and development of pancreatic ductal adenocarcinoma by modulating Lin28b [121,122]. In addition, SIRT6 is also an oncogene that promotes cell proliferation and survival by enhancing COX-2 expression in skin cancer [72]. SIRT6 overexpression in NSCLC is linked to a poor prognosis but is conducive to metastatic and chemotherapeutic resistance [122,123].

Some reports have indicted SIRT7 as an important regulator of TGF-β signaling and an inhibitor of breast cancer metastases and that its deficiency can promote the metastasis of breast cancer cells [85]. SIRT7 deacetylates and enhances β-TrCP1-mediated SMAD4 degradation. At the same time, a deficiency in SIRT7 both activates TGF-β signaling and intensifies EMT. Similar observations have demonstrated that SIRT7 expression is decreased in OSCC cell lines and human OSCC/OSCC tissues with lymph node metastasis [81]. Its overexpression not only suppresses the expression of E-cadherin but also suppresses the expression of mesenchymal markers, lowers the level of acetylated SMAD4 in OSCC cells and hinders OSCC lung metastasis. It is thus notable that SIRT7 drives SMAD4 deacetylation to suppress EMT in OSCC metastasis [81].

## 5. Sirtuins in Tumorigenesis

The role that sirtuins play in cancer has been a subject of debate. Because they are able to both promote and suppress tumorigenesis, sirtuins may act as a double-edged sword in cancer (Figure 2 and Table 1). SIRT1 is highly expressed in several cancers, including prostate carcinoma, acute myelogenous leukemia, colon cancer, and some nonmelanoma skin cancers [124,125,126,127,128]. However, the expression of SIRTl is also suppressed in many other cancers, such as glioblastoma, bladder cancer, and ovarian cancer [129]. The two opposite functions of SIRT1 have been reported not only in tumor promotion and inhibition but also in tumor development. It can serve as either an oncogene or as a normal epigenetic regulator. Its role relies on the oncogenic pathway specific to particular tumors because of complexity [30]. As shown in previous studies, SIRT1 functions in tumorigenesis through its antiapoptotic activity, where it deacetylates proapoptotic proteins and helps cells survive under genotoxic and oxidative stresses [18,30,130].

Regarding SIRT2, knockout (KO) studies have revealed that the loss of *Sirt2* leads to the development of tumors earlier in KO mice than in wild-type (WT) mice [39]. Despite not discovering the cancer-prone phenotype in *Sirt2* KO mice, Serrano et al. found an increase in tumorigenesis in KO mice when attacked by carcinogens [131]. SIRT2 may be a weak tumor suppressor in carcinogenesis, as mentioned above. Nevertheless, Jing et al. found that inhibiting SIRT2 results in broad anticancer activity in a variety of cancer cell lines and mouse models of breast cancer [132]. Its anticancer effect is related to the decrease in the MYC level because SIRT2 inhibition promotes MYC ubiquitination and degradation. In normal cells, there may be several factors that exert tumor-inhibiting activity, which is needed for the growth and survival of transformed cells [132].

SIRT3 plays a conflicting role not only in different types of cancer, such as gastric cancer [133,134], lung cancer [49,135,136], and colon cancer [137,138,139,140], but also in malignancies originating from the same types of tissue. SIRT3 has been found to affect tumorigenesis by depleting reactive oxygen species (ROS), modulating metabolism, and regulating proliferative or apoptotic pathways [141]. On the one hand, SIRT3 functions as a tumor suppressor, decreasing tumorigenesis by suppressing glycolysis proliferation and its downstream transcriptional activity under hypoxic conditions [142]. SIRT3 knockdown, a process that can be depressed by treatment with the antioxidant N-acetyl cysteine, drives tumorigenesis in xenograft models, whereas SIRT3 overexpression impedes tumorigenesis in xenografts [143]. Moreover, SIRT3 can also function as a tumor promoter. By deacetylating and activating lactate dehydrogenase, SIRT3 facilitates anaerobic glycolysis and carcinogenesis in gastric cancer cells [133]. In summary, the role of SIRT3 in tumorigenesis remains a matter of debate.

SIRT4 acts as a tumor suppressor in liver cancer, breast cancer and colorectal cancer [144,145,146]. *Sirt4* KO mice can be spontaneously infected with lung cancer, liver cancer, breast cancer, and lymphomas [56]. Low SIRT4 expression is associated with poor pathological grading and other clinical and pathological parameters in gastric, colon, liver, lung, and esophageal cancers [94]. Similarly, low levels of the SIRT4 protein are correlated with a poor prognosis in colon, lung, and esophageal cancers [94]. However, SIRT4 has not been proved to act as a tumor suppressor gene [147,148]. It may also play an oncogenic role in the tumors and conditions mentioned above. However, such a role for SIRT4 requires further investigation.

Only a limited amount of research has been conducted on SIRT5 in tumorigenesis. Several recent studies have shown that SIRT5 may play a tumor-promoting role in multiple types of cancer, such as HCC [65], colon cancer [63], human osteosarcoma [63] and breast cancer [149]. Moreover, the SIRT5 gene frequently shows an increase in duplication in specific cancer types, including uterine cancer, breast cancer, cutaneous and uveal melanomas, lung cancer, and lymphoma [150]. However, high SIRT5 expression is interrelated with a favorable prognosis for patients with HCC; the downregulation of SIRT5 is correlated with high ACOX1 succinylation and activity and poor survival in HCC patients [151]. Clearly, further studies are required to examine the possible involvement of SIRT5 in tumorigenesis.

SIRT6 also acts as a double-edged sword in cancer. In most cases, it functions as a tumor inhibitor, functioning to prevent genomic instability, maintain telomere integrity, and regulate metabolic homeostasis [152]. However, accumulated data have suggested its oncogenic role in different types of cancer [122,123]. Therefore, it would be interesting to probe the mechanism involved in its negative regulation [152].

SIRT7 may promote tumorigenesis in human cancer. Previous research has shown that SIRT7 plays the role of a tumor promotor in various cancers, such as epithelial prostate carcinoma, gastric cancer, hepatic cancer, cholangiocarcinoma, ovarian cancer and breast cancer [82,84,153,154,155]. Although SIRT7 depletion markedly weakens the tumorigenicity caused by human cancer cell xenografts in mice, SIRT7 itself does not give rise to oncogenic transformation of primary fibroblasts [156]. Therefore, the tumor-promoting performance of SIRT7 may be a secondary effect most likely due to its positive impact on ribosome biogenesis [157].

## 6. Sirtuins and Cancer Immunotherapy

Immunotherapy has arisen as feasible alternatives in the treatment of cancers following the clinical success of immune checkpoint inhibitors [158]. Immune checkpoint inhibitors have some better efficacy in treatment of different kinds of cancers, including melanoma, non-small-cell lung cancer and renal carcinoma [159]. PD-L1 can be transcriptionally regulated by NF-kB, and inhibition by HDAC inhibitor. The nuclear factor-kB (NF-kB) signaling plays a major role in inflammation and immunity, which regulates the expression of cytokines, chemokines and other pro-inflammatory agents [160]. Although few reports showed sirtuins play important roles on immunotherapy, sirtuins, as deacetylases are central to immunity. Several sirtuin family members, such as SIRT1, SIRT2, and SIRT6, can regulate NF-kB-driven immune responses through the protein deacetylation. Recent research demonstrated that SIRT7 can inhibit the expression of PD-L1 though reducing acetylation of MEF2D in hepatocellular carcinoma cells not exposed to interferon gamma [161]. The PD-L1 expression of cancer cells can bind to PD1 on CD8^+^ T cells, which may prevent T cell proliferation and reduce their anti-tumor immunity response. Strategies to manipulate the activity of SIRT7 may improve the efficacy of immune therapies for hepatocellular carcinoma. Furthermore, sirtuin modulators, including activator and inhibitor, have anti-tumor capability (Table 2). As sirtuin activators, both resveratrol and piceatannol could upregulate the expression of PD-L1 though HDAC3/p300-mediated NF-κB signaling in breast and colon cancer cells, which combined with anti-PD-L1 immunotherapy may reap clinical benefits in no or low PD-L1 level cancer patients [159]. However, there are also few reports that sirtuin modulators are involved in immunotherapy. We firmly believed that sirtuins could make an important contribution to anti-tumor immunity response and their modulators could improve the efficacy of immunotherapy. Therefore, further research is needed to better understand the roles of sirtuins on anti-tumor immunity.

## 7. Conclusions and Perspectives

Proteins of the sirtuin family play a role in both normal and pathological conditions that are closely related to tumors in several different pathological processes, including tumor cell proliferation, apoptosis, metastasis, and tumorigenesis. Of these proteins, SIRT1, SIRT3, and SIRT6 play a dichotomous role in different types of cancer depending on the type, stage and microenvironment of the tumor. SIRT2 and SIRT4 have been reported to protect against cancer. SIRT5 and SIRT7 play a tumor-promoting role, as they are overexpressed in human cancer and are also associated with unsatisfactory outcomes. Therefore, unravelling the underlying mechanisms and conditions that allow these proteins to play two opposite roles in cancer is perhaps one of the main challenges in cancer treatment.

Acetylation is a process of transcriptional modification that plays an important role in the regulation of protein interactions, protein catalytic activity, and stability, and thus in physiological functions, including proliferation, apoptosis, and metastasis. SIRT1 to SIRT3 have strong deacetylase activity. SIRT1, as the most well-investigated member of the sirtuin family, is multifaceted in regulating cancer progression depending on its substrate proteins NF-kB, P53, KU70, HIFs, and so on. The substrate proteins of SIRT2 include histone H4, α-tubulin, β-catenin, P53, FOXO1, and PEPCK1, which regulate biological functions by regulating the deacetylation of these proteins. SIRT3, as the major mitochondrial deacetylase, mainly promotes mitochondrial metabolism and inhibits the production of ROS. In addition, SIRT3 can bind to and deacetylate the F-box protein Skp2, rendering it unstable, while Skp2 refers to a protein that serves to promote tumorigenesis via the ubiquitination and degradation of tumor suppressors. The deacetylase activity of SIRT4 to SIRT7 is considered weak or even difficult to detect; SIRT4 mainly exerts ADP-ribosyltransferase activity. SIRT5 is another member of the sirtuin family found in mitochondria. It works to mediate FOXO3 deacetylation, a crucial characteristic to protect lung epithelial cells from the apoptosis induced by cigarette extract. SIRT6 can deacetylate H3K9 at the HIF1α and MYC promoters to modulate cell proliferation. SIRT7 is a nuclear silencing regulatory protein mostly located in nucleoli. SIRT7 is also a specific deacetylase of H3K18 that regulates the biological processes of ribosomes by controlling the synthesis of rRNA, tRNA and ribosomal proteins. In addition, SIRT7 can be acquired by particular transcription factors, such as ELK4 and MYC, and can inhibit gene expression by deacetylating H3K18. Interestingly, SIRT7 can attenuate the expression of PD-L1 though reducing the acetylation of MEF2D. These findings suggest that sirtuin protein family may involve in immune-regulatory activity in cancer cells, which can provide new ideas for cancer immunotherapy.

With the development of molecular biology, the sirtuin family has gradually become the target of disease prediction and of tumor treatment. Thus, developing specific activators or inhibitors of these sirtuins might reveal a large number of therapeutic opportunities (especially immunotherapy) for different types of cancer. Histone deactylase (HDAC) inhibitors, sirtuin inhibitors/activators of the same deacetylase, have been listed on the market but are still at the development phase. Thus, for each sirtuin protein, its mechanism of action requires a great deal of comprehensive research before it is profitable. It is possible to intervene in disease or tumor progression with small molecules, either natural or synthetic. In some pathological mechanisms, such as breast cancer and liver cancer, sirtuin family proteins are upregulated so significantly that efficient inhibitors are in urgent demand. Most of the sirtuin inhibitors reported, such as nicotinamide, Ex-527 and the like, are competitively inhibitive with high selectivity. Furthermore, recent research have shown that several sirtuin activators (such as resveratrol and piceatannol) could upregulate the expression of PD-L1 in breast and colon cancer cells, which may exert more clinical benefits though co-administering with anti-PD-L1 immunotherapy.

In summary, recent studies have noted the contribution of sirtuins to the fight against cancer. Further explorations on sirtuins should evaluate the following: (1) The dichotomous roles should be further elucidated in different types of tumor cells, tumor tissues, and metastases. (2) Sirtuins are equipped with various enzymatic activities, such as deacetylase and ribosyltransferase activity. Do they act alone or in concert? Which play a regulatory role? How to involve in anti-tumor immune-regulatory activity? (3) What are the potential side effects caused by sirtuin activation and inhibition? How can sirtuins be maintained at appropriate levels to inhibit the progression of cancer cells? (4) How can more targeted and nontoxic enzyme inhibitors or activators be designed and synthesized to treat cancer? Do they improve clinical benefits when co-administered with anti-tumor immunotherapy? (5) What are the relationships between the expression of sirtuins and cancer? What relationships are helpful in identifying the population with a high risk of tumorigenesis and metastasis? What relationships are helpful in finding better ways to prevent, diagnose and treat cancers? Studies on the roles of sirtuins in cancer progression have deepened our understanding of tumorigenesis so significantly that advancements might give rise to novel therapeutic strategies. Despite their promising future, a very large amount of work is required prior to considering sirtuin proteins as valuable therapeutic targets in the clinic. Hopefully, the present review will contribute to the development of this field.

## Figures and Tables

**Table 1:** Roles of sirtuins in different kinds of cancers.

Name	Function	Promoter	Genes or Pathways Involved	Suppressor	Signaling Pathways Involved
SIRT1	Viability	TC [22]	MYC	BC [21]	BRCA1
		CRC [23]	Oct4, Nanog, Cripto, Tert and Lin28	NSCLC [31]	K-RAS; PI3K
		Leukemia [24,25]	STAT5 signaling; FOXO1, p53, Ku70		
		RB [28]	Rb, p107 and p130		
		Glioma [32]	EMT pathway		
		BC [33]	AKT		
	Apoptosis	PC [30]	P53		
		NSCLC [31]	K-RAS; PI3K		
		BC [21,30]	BRCA1, surviving; P53; Rb		
	Metastasis	PC [17]	ZEB1, EGF signaling, EMT pathway	BC [20]	Smad4/β-catenin
		GC [34]	DBC1	NSCLC [31]	K-RAS; PI3K
		Glioma [32]	EMT pathway		
	Tumorigenesis	GC [34]	DBC1	BC [21]	RCA1, surviving
		BC [35]	AKT		
SIRT2	Viability	HCC [36]	α-tubulin	OC [37]	CDK4
		NB [38]	MYCN	HCC [39]	APC, CDC20
		PAC [38]	MYC	BC [39]	APC, CDC20
	Apoptosis			NSCLC [40]	P53
				CCA [41]	MYC
	Metastasis	HCC [42,43]	AKT/GSK3β/β-catenin Axis		
		GC [44]			
	Tumorigenesis	BC [45]	Slug	HCC [39]	APC, CDC20
				BC [39]	APC, CDC20
SIRT3	Viability	BLC [46]	P53	PC [47]	MYC; PI3K/AKT pathway
		CRC [48]	AKT/PTEN	NSCLC [49]	Bax/Bcl-2, P53
				HCC [50]	PI3K/AKT pathway
				PDC [51]	
	Apoptosis	NSCLC [49]	Bax/Bcl-2, P53	OSCC [52]	RIP
		Leukemia [53]	AKT, Bax/Bcl-2		
	Metastasis	CRC [48]	AKT/PTEN	HCC [50]	PI3K/AKT pathway
				PDC [51]	
				PC [54]	FOXO3A, Wnt/β-catenin pathway
				OC [55]	Twist
	Tumorigenesis	OSCC [53]	RIP		
SIRT4	Viability			OC [56]	GDH
				NS NSCLC [57]	ERK/Drp1 Axis
				ESCC [58]	GDH
				CRC [59]	
				GC [60]	
	Apoptosis				
	Metastasis			NSCLC [57]	ERK/Drp1 Axis
				ESCC [58]	GDH
				CRC [59]	E-cadherin
				GC [60]	E-cadherin
				HCC [61]	LKB1/AMPKα/mTOR axis
	Tumorigenesis			NSCLC [57]	ERK/Drp1 Axis
				HCC [61]	LKB1/AMPKα/mTOR axis
SIRT5	Viability	CRC [62,63]	GLUD1, SHMT2		
		OSA [63]	SHMT2		
		NSCLC [64]	PKM2		
		HCC [65,66]	E2F1		
		RCC [67]	SDHA		
	Apoptosis			NB [68]	
	Metastasis	HCC [65,66,69]	E2F1, Vimentin		
	Tumorigenesis	CRC [62]	GLUD1		
		NSCLC [64]	PKM2		
		RCC [67]	SDHA		
SIRT6	Viability	HCC [70]	Bax	HCC [71]	PKM2
		SSCC [72]	COX-2, AKT, AMPK	CRC [73]	PTEN/AKT signaling
				ACC [74]	NF-κB signaling
				GBM [75]	JAK2/STAT3 pathway
	Apoptosis	CRC [73]	PTEN/AKT signaling	HCC [70]	Bax
		GBM [75]	AK2/STAT3 pathway	FSA [76]	NF-κB signaling
		HCC [77,78]	ERK1/2 pathway	CC [76]	NF-κB signaling
	Metastasis			HCC [71,79]	PKM2
				CRC [73]	PTEN/AKT signaling
				ACC [74]	NF-κB signaling
	Tumorigenesis			HCC [71,79]	PKM2
				ACC [74]	NF-κB signaling
SIRT7	Viability	OSA [80].	CDC4	OSCC [81]	SMAD4
		OC [82]	NF-κB		
		BC [83]	p38-MAPK		
		GC [84]			
	Apoptosis			OC [82]	NF-κB
				BC [83]	p38-MAPK
				GC [84]	
	Metastasis	OSA [80]	CDC4	OSCC [81]	SMAD4
		OC [82]	NF-κB	BC [85]	TGF-β signaling
		BC [83]	p38-MAPK		
		GC [84]	Bax/Bcl-2		
	Tumorigenesis	OSA [80]	CDC4		

**Note:** ACC: Adrenocortical carcinoma; BC: Breast cancer; BLC: Bladder cancer; CC: Cervical cancer; CCA: Cholangiocarcinoma; CRC: Colorectal cancer; ESCC: Esophageal squamous cell carcinoma; FSA: Fibrosarcoma; GC: Gastric cancer; GBM: Glioblastoma multiforme; HCC: Hepatocellular cancer; NB: Neuroblastoma; NSCLC: Non-small cell lung carcinoma; OC: Ovarian carcinoma; OSCC: Oral squamous cell carcinoma; OSA: Osteosarcoma; PAC: Pancreatic cancer; PC: Prostate cancer; PDC: Pancreatic ductal cancer; RB: Retinoblastoma; RCC: Renal cell carcinoma; SSCC: Skin squamous cell carcinoma; TC: Thyroid cancer.

**Table 2:** Selected sirtuin modulators (Activator and inhibitor).

No.	Name	Roles	Modulated Targets	Biological Actions for Cancers
1	Resveratrol	Activator	SIRT1, SIRT3, and SIRT5 [162,163]	Inducing autophagy in lung cancer cells [164]; inducing apoptosis and upregulation of PD-L1 expression in breast and colon cancer cells [159].
2	Piceatannol	Activator	SIRT1, SIRT3, and SIRT5 [162,163]	Inducing apoptosis and upregulation of PD-L1 expression in breast and colon cancer cells [159]; inhibiting migration and invasion in prostate cancer cells [165].
3	SRT2104	Activator	SIRT1 [166,167]	No report
4	UBCS039	Activator	SIRT5 and SIRT6 [168]	Inducing autophagy-associated cell death in cervix, colon and lung cancer [169].
5	Ex-527	Inhibitor	SIRT1 [170]	Inducing growth inhibition and apoptosis in lung cancer cells [171].
6	UBCS0137	Inhibitor	SIRT2 [172]	No report.
7	ELT-11c	Inhibitor	SIRT1, SIRT2, and SIRT3 [173]	No report.
8	Nicotinamide	Inhibitor	SIRT1, SIRT2, SIRT3, SIRT5, and SIRT6 [24,174,175]	Reducing inflammatory macrophages and promoting skin cancer chemoprevention [176]; Chemoprevention of breast cancer recurrences [177].

## Abbreviations

ADP: Adenosine diphosphate; AML: Acute myeloid leukemia; AP-1 transcription factor subunit; β-catenin: Catenin beta 1; CDK: Cyclin dependent kinase; COX-2: Cytochrome c oxidase subunit II; CtBP: C-terminal Binding Protein; Drp1: Dynamin related protein 1; E2F1: E2F transcription factor 1; FOXO3: Forkhead box O3; GC: Gastric cancer; GLUD1: Glutamate dehydrogenase 1; GSK-3β: glycogen synthase kinase 3 beta; H4K16: Histone H4 at lysine 16; HCC: Hepatocellular carcinoma; HIF1α: Hypoxia inducible factor 1 subunit alpha; IDH2: Isocitrate dehydrogenase 2; JUN: Jun proto-oncogene; KDM4A: Lysine demethylase 4A; KU70: XRCC6; MEF2D: myocyte enhancer factor 2D; MMP: Matrix metalloproteinase; NAD: Nicotinamide adenine dinucleotide; NF-κB: Nuclear factor kappa B subunit 1; NMNAT2: Nicotinamide nucleotide adenylyltransferase 2; NSCLC: Non-small-cell lung cancer; P53: Protein 53; P65: Protein 65; PD-1: programmed cell death 1; PD-L1: programmed cell death ligand 1; PDHA1: Pyruvate dehydrogenase E1 alpha 1; p-ERK1/2: Phosphorylation of extracellular signal-regulated kinase 1/2; PKM2: Pyruvate kinase M2; RB1: RB transcriptional corepressor 1; SHMT: Serine hydroxymethyltransferase; SIRT: Sirtuin; aa: amino acid; SOD1: Cu/Zn superoxide dismutase 1; TGF-β: Transforming growth factor-β; X-ray repair cross complementing.
